# Recognizing and Responding to Anti-Science in Environmental and Public Health Research and Practice

**DOI:** 10.3390/ijerph20042927

**Published:** 2023-02-08

**Authors:** John Øvretveit

**Affiliations:** 1 Medical Management Centre, Department of Learning Management Informatics and Ethics, Karolinska Institutet, Region Stockholm, 17176 Stockholm, Sweden; jovretb@aol.com; 2 Stockholm Health Care Services, Region Stockholm, 17176 Stockholm, Sweden

**Keywords:** anti-science, narrative research, behavioral science, implementation

## Abstract

This perspectives article considers the challenges posed by anti-science and how we can use research to respond more effectively. In public health, the challenges were more visible and the impact more serious during the COVID-19 pandemic. In part, this was due to a more organized anti-science and effective use of narrative methods. Regarding climate change, the role of anti-science represents a critical issue, but perhaps more recognized in environmental research and practice. The article draws on a narrative review to show some of the research into the nature of anti-science and the challenges it poses. It proposes that, as researchers, practitioners, and educationalists, we can be more effective if we make more use of recent research in the sciences of communications, behavior, and implementation, and shows some of the resources we can use to help our work be more relevant in the new era in which we are living.

## 1. Introduction

*“All lies and jest, still a man hears what he wants to hear and disregards the rest”*

Although there is debate among researchers about the criteria for good quality evidence, most researchers with a cancer diagnosis would decide treatment after considering the evidence. With respect to environmental and public health sciences, there is perhaps less consensus and more uncertainty about evidence. In the early phases of the COVID-19 pandemic, many studies reported evidence that was questionable, even if some was better than nothing. Examples are early knowledge about viral transmission in environmental health studies and studies on vaccine durability.

Anti-science is a set of attitudes, an ideology, and a movement that involve a complete rejection of science and the scientific method [1]. The purpose of this commentary is to encourage researchers, practitioners, and educationalists to take anti-science more seriously and use effective communication to be more relevant in the new world. We need to define more precisely the object of concern: is it what seems to be a growing public indifference to scientific evidence, is it public confusion about the evidence, or is it the heterogeneous anti-science “movement”, or all of these and more? This knowledge is needed to develop both a “science of anti-science” in our fields and more serious and effective counteractions. This requires conceptual analysis and methods of measurement to study the phenomena and to develop and evaluate interventions and strategies. There is good material from the environmental and climate change fields to help us, material which recognizes the powerful strategies of the anti-science messengers and the changing psychology and attitudes of segments of the public and media [2].

Anti-science poses threats to and beyond the academic community. Many researchers and educationalists feel that anti-science is an attack on their values and their vocational ethic to seek and teach scientific truth. Perhaps, those feeling threatened the most are those who see a devaluation of positivist and experimentalist scientific methods. Some of my clinical research colleagues are concerned about movements within the academic community criticizing the traditional hierarchy of evidence, and even the relevance of the experimentalist method when real world evidence can be more robust with the skillful use of machine learning and artificial intelligence [3]. A few take the view that these criticisms strengthen anti-science at a time when more unity among all researchers and academics is needed.

This commentary is not the place to discuss what is and is not science, but this centuries-old debate is relevant to the point of the commentary. This debate includes the criticisms of “scientism” and of scientific methods and attitudes being applied to questions that do not yield fruitful answers, and to an over-extension of the scientific approach to all areas of human life [4]. The simple and inadequate approach I have taken to side-step the “what is scientific evidence” debate is to view evidence as what is published in a peer-reviewed academic journal. There have certainly been developments over the last three years within and related to academia which have devalued rigorous research, including public “expert” proclamations by academics. My own experience recently was of a journal editor sending my paper to academic reviewers for review, an act which, without me knowing it, triggered an automatic system through which the publishing company sent the paper to another company that published it as a preprint. When the paper was subsequently rejected, and for good reasons, I revised and resubmitted it to another journal with another publishing company. This journal rejected immediately because parts were “already published”, having been identified by that journal’s automatic plagiarism software. This was one of many events over the last three years that have undermined how much I used to value both published research and academic colleagues in general, but it has also usefully strengthened my resolve to do more through independent assessments of the studies, which is all for the best, but may not be feasible for the general public and many practitioners.

The now more publicly visible lack of consensus within the academic community about what constitutes valid science can undermine what has been a major public belief in science and can be exploited by anti-science proponents. But perhaps this is not the main threat posed by anti-science, as it has already achieved a powerful influence without needing to reference conflicting “academic expert” public pronouncements. Possibly, the real influence of anti-science is due to both listeners being more ready to hear the message and those taking this perspective using effective methods to influence public views, principally storytelling, social media communication, and peer transmission and amplification. Research shows these are effective for both changing attitudes and behavior, yet most researchers have made little use of these methods to communicate or implement their findings or counter the messages and perspective of anti-science promulgators. I believe we have a moral and professional duty to counter anti-science with these and other methods and to make more use of effective communications and behavior change methods, but will need to do so in a skillful and research-informed way. This has implications for research, education, practice, and the policy of our academic institutions: we need to catch up with the new world in which we are living. This additional burden is not a popular message when most of us are already facing increased work and complexity in our research, education, and practice.

## 2. Facing the Challenge with Evidence-Based Strategies

Those advancing an anti-science perspective make effective use of storytelling as a strategy. From the beginning of language, people have produced and reproduced learning and culture through stories. There is a reason why all religions use stories: because they work. In modern times, an often-heard phrase in journalism is “what is the story here?”, with one textbook describing the occupation of journalism as “storytelling with a purpose” [5]. Short story examples from peoples’ lives are often used as an introduction to media reports to engage the audience, or later on to elaborate points in the report. Similarly, the field of marketing uses systematic approaches to develop stories so as to influence target consumers [6]. Other business research reports that “narrative leadership” is an important way to carry out change strategies [7]. A large industry has developed to train leaders to “lead through stories”. In one study, an example is given of a biotechnology leader seeking investor finance by not commencing the presentation with market projections and the business plan, but rather with an introductory story: “he could captivate them by telling the story of his father, who died of a heart attack, and of the CEO’s subsequent struggle against various antagonists—nature, the FDA, potential rivals—to market the effective, low-cost test that might have prevented his father’s death” [8].

In our field, some public health programs have found success by using storytelling in their strategies [9]. A review of research in health promotion notes that stories are increasingly used to help the uptake of research evidence [10]. This review provides a useful framework to guide the designing of stories for practitioners that is based on the study findings. The authors describe how to decide whether versions of this method are appropriate or feasible to encourage innovation, how to decide methods to specify goals and audiences, and how to develop and test a storytelling intervention. The framework gives guidance for researchers to develop an intervention by working with the target groups to formulate messages or scripts and decide the best story-delivery medium [10].

Public health and global health have long recognized challenges associated with successfully achieving uptake of evidence-based strategies or interventions in different communities; examples include vaccination uptake and low-cost high-nutrition diets. An approach that has shown some success in difficult-to-reach communities is involvement of community workers or peers from the same communities, perceived by their target populations as “people like us, who speak like us” and who are often perceived as more trust-worthy than other individuals. Another successful approach is the designing of interventions together with community representatives that are tailored for specific groups. One example is a project that found how collaboration with community leaders over time was needed to then carry out co-design of communications to provide COVID-19 information to communities with diverse backgrounds [11].

Researchers are increasingly being required to use evidence-based approaches to better communicate their findings to funders and other authorities. A common approach is to use both traditional and social media methods. A key issue for environmental and public health researchers is to influence policy. Policy research suggests that there are different ideas amongst policy makers about what constitutes good evidence and that policy makers often do not control policy-making in the way that a rational model would envisage. [12]. Research was used to propose three methods: “tell a story, since evidence will not speak for itself”, “form coalitions and provide evidence to empower your allies (do not expect everyone to be influenced by the evidence)” and “be flexible, since a successful framing strategy in one venue may fail in another” [13].

Do stories devalue or add value to academic reports? For academic audiences, are we too afraid they will be viewed as unscientific anecdote and as a trick to divert attention from a critical appraisal of the evidence of the study? Experience and evidence noted above suggests that, for all audiences, we need to appeal to the heart rather than to the mind alone, combining stories when presenting a research evidence report. Using storytelling can be effective for health-promoting behavior change and to help reduce inequities within groups other than academics. Evidence, including neurological scanning, is showing that stories designed using research-informed guidance with the best story-teller for the audience in question connects with peoples’ emotions. This can be effective to trigger action.

## 3. Conclusions

The threat of anti-science to our work as researchers, practitioners, and educationalists in environmental health and public health has become clearer in recent years. Climate change and public health challenges have increased at the same time as anti-science sentiments have become more widespread and the movement itself more effective. The future significance of anti-science is high, and, perhaps, has not been sufficiently recognized as a subject for environmental and public health research and as an issue to address in the communication and implementation of research. Some evidence-based approaches that can be used include the designing of systematic narratives, the application of recent behavioral research on the subject, and the development of partnership research approaches and co-designing of implementation strategies. Using and conducting more research on the subject could make environmental and public health research and practice more effective and relevant to the new era in which we are now living.
